# Nurses’ Perspectives on the Implementation of Knowledge in Clinical Practice: A Qualitative Study

**DOI:** 10.3390/healthcare14050555

**Published:** 2026-02-24

**Authors:** Raquel Sofia Neves da Silva, Óscar Ramos Ferreira, Inês Agostinho, Raimunda Silva, Maria Helena Barbosa, Patrícia Braga, Mara Quaglio Chirelli, Cristina Lavareda Baixinho

**Affiliations:** 1 Nursing Research Innovation and Development Centre of Lisbon (CIDNUR), School of Nursing, Universidade de Lisboa, 1900-160 Lisboa, Portugal; 2 Graduate Program in Nursing and Collective Health, Universidade de Fortaleza-Unifor, Fortaleza 60811-905, CE, Brazil; 3 National Council for Scientific and Technological Development–CNPq, Nursing Department at Hospital Care (DEAH), Federal University of Triangulo Mineiro (UFTM), Uberaba 38025-180, MG, Brazil; maria.barbosa@uftm.edu.br; 4 Center for Studies on Children and Adolescents (NECA), Federal University of São João Del-Rei, Divinópolis 35501-296, MG, Brazil; 5 Health Education Research Group/CAPES-CNPq, Marília Medical School, Marília 17519-030, SP, Brazil; 6 Center for Innovative Care and Health Technology (ciTechcare), Polytechnic of Leiria, 2414-016 Leiria, Portugal

**Keywords:** evidence-based practice, nursing, students, leadership

## Abstract

**Highlights:**

**What are the main findings?**

- A shift in organisational culture is essential to support evidence-based practice.
- Teams need to be empowered to engage in evidence-informed decision-making.

**What are the implications of the main findings?**

- Sustaining evidence-based practice in nursing remains challenging, underscoring the need to strengthen the production, dissemination, and application of knowledge, promote consistent practice standards, and support the ongoing development of nurses’ competencies.
- Ensuring protected time for nurses to critically compare current practice (“ways of doing”) with evidence-based recommendations and translate them into routine care.

**Abstract:**

**Background**: The delivery of nursing care based on available evidence and centered on the individuals who require it and has a positive impact on the quality of professional interventions, leading to health benefits for the population across all core domains of nursing practice, as well as contributing to the advancement of the profession. **Objective:** To analyze nurses’ perspectives on the effects of their participation in evidence implementation programs in clinical nursing. **Methods:** In the Qualitative Descriptive Study, the study participants were nurses who took part in the “Safe Transition” project, a collaborative initiative involving three institutions: a nursing school, a hospital, and a network of primary healthcare institutions. The data were collected through semi-structured interviews that were analyzed by two researchers using content analysis techniques, with the support of the qualitative data analysis software. **Results:** From the content analysis of semi-structured interviews conducted with 17 nurses involved in knowledge implementation projects in clinical practice, the following categories emerged: Cultivating a spirit of inquiry and an evidence-based practice culture; Critically appraising established practices and evidence-based recommendations; and Integrating evidence into clinical expertise to drive change, improve outcomes, and enhance the quality of care. **Conclusions:** Effective communication and structured opportunities for knowledge sharing emerged as central to the critical examination of clinical practice and to the development of professionals’ competencies in evidence use. Evidence implementation was further motivated by professionals’ recognition that it can generate tangible benefits for healthcare service users. Collectively, these findings inform recommendations for clinical practice, nursing education, and future nursing research.

## 1. Introduction

The provision of healthcare based on the best available evidence and centered on the individual is a universal recommendation to ensure the quality and cost-effectiveness of interventions [1]. The use of evidence in clinical practice is a complex process that requires a shift in organizational culture to build evidence ecosystems [2], as its application has been globally recognized as a key driver for improving healthcare services [3].

The study by Ominyi and colleagues highlights the need for a multilevel strategy that strengthens leadership development, tackles organizational barriers, and addresses cultural resistance, while explicitly linking these actions to measurable improvements in evidence-based nursing practice [4].

Healthcare organizations must recognize that successful Evidence-Based Practice (EBP) implementation depends on aligning leadership behaviors with systemic constraints, thereby creating sustainable environments for evidence-informed care. In addition, overcoming organizational constraints requires deeper integration of EBP into existing workflows to support long-term sustainability [4].

Other authors support these recommendations, emphasizing that it is essential for nurses to build their body of knowledge, standardize practice, and improve patient care outcomes [1,5] to ensure equitable and efficient service delivery [1]. Although the discussion surrounding EBP is not new within the healthcare debate, researchers frequently observe that evidence is applied inconsistently and ineffectively in clinical practice [5,6,7].

The study conducted by Sulaiman et al. emphasize the challenges nurses encounter in integrating research into clinical practice and highlight the need for ongoing efforts to promote the utilization of EBP and research [8], because its application remains suboptimal [4,8,9,10]. This shortfall is attributed to gaps in knowledge and competencies required to read, analyze, synthesize, and implement research-based interventions [3,7], insufficient time to conduct evidence synthesis studies and implement corresponding programs, difficulties in comprehending research studies and guidelines [3], as well as a lack of financial, material, and human resources [2,3,4]. Additionally, limited organizational support [2] and inadequate familiarity with research methods and techniques [3,7] further hinder the effective adoption of EBP.

Gaps in nursing undergraduate curricula regarding research and EBP, along with the lack of opportunities for students to engage in research and EBP projects that would allow them to develop competencies in this field, further hindering the adoption of EBP [7,11]. A recent study aimed at identifying the factors influencing the implementation of EBP concluded that the lack of autonomy among nurses is the most significant barrier to their ability to apply research findings in practice and, consequently, to adopt EBP [3].

Tucker and Gallagher-Ford [12] argue that among the seven steps of EBP (Cultivating a spirit of inquiry and an EBP culture, Formulating clinical questions, Searching for the best available evidence, Critically appraising the evidence and recommending practice changes, Integrating the evidence with clinical expertise and patient/family preferences and values, Evaluating the outcomes of practice decisions or changes based on evidence and Disseminating the results of the EBP change), the implementation phase (step 4), where evidence is integrated with clinical expertise and patient/family preferences and values, remains the most challenging [12].

The solution to overcoming some of the difficulties, particularly in terms of the implementation of programs, may lie in the use of educational materials, reminders, the adoption of decision support systems, performance feedback, the involvement of leaders who train and assist in decision-making, and multidisciplinary collaboration [12]. The incorporation of EBP into nursing curricula, as well as the existence of national and organizational policies that promote the use and implementation of EBP programs, are crucial [7,11], and should simultaneously foster belief in the value and benefits of EBP while enhancing perceived self-confidence in knowledge [10].

Considering the above, the objective of this study is to analyze nurses’ perspectives on the effects of their participation in evidence-based practice implementation programs in nursing practice.

## 2. Materials and Methods

### 2.1. Study Design

To address the research question, ‘What is the perspective of nurses regarding the effects of an evidence implementation program in clinical practice?’, we opted for a Qualitative Descriptive Study [13]. This type of study is rooted in the fundamental principles of naturalistic inquiry [13]. Qualitative studies facilitate the advancement of knowledge in healthcare, as they explore complex phenomena whose wholeness, beyond their depth, is also contingent on their intensity [14]. They enable a deeper understanding of individuals’ beliefs, experiences, attitudes, behaviors, and interactions, bringing to science a more profound comprehension of participants’ experiences and engagement in research [13,14]. In preparing this article, we followed the COREQ (Consolidated Criteria for Reporting Qualitative Research) recommendations [15].

### 2.2. Sample and Recruitment

The study participants were nurses who took part in the “Safe Transition” project, a collaborative initiative involving three institutions: a nursing school, a hospital, and a network of primary healthcare institutions. One of the project’s primary objectives was to implement evidence-based practice (EBP) in clinical settings. The initiative included nursing faculty members, final-semester undergraduate nursing students (4th year, 2nd semester), and clinical nurse preceptors responsible for mentoring the students. As an institutional project, it required a joint effort among partners to define the activities aimed at enhancing EBP within healthcare services and integrating scientific knowledge into clinical practice.

This study focused on the nurses’ participation in the project, with the following predefined inclusion criteria: a minimum of three years of involvement in the “Safe Transition” project, at least five years of professional experience at the time of their enrolment, an active role as clinical preceptors for nursing students, and engagement in knowledge implementation activities. A total of 24 participants met these criteria. No a priori sample size was specified. Instead, participants recruitment and data collection were guided by the principle of thematic saturation. Saturation was considered to have been achieved by the 13th interview, when further interviews yielded no new themes. To enhance the credibility and robustness of this assessment, four additional interviews were conducted to confirm that saturation had been reached.

### 2.3. Data Collection

The data were collected through semi-structured interviews conducted between April and November 2024. Initially, participants were contacted by one of the researchers to ensure they met the predefined eligibility criteria and to assess their willingness to participate. In the subsequent phase, an informed consent form was sent via email for review, signature, and return to the principal investigator. Following this, the interviews were scheduled.

Based on the research question, What is the perspective of nurses regarding the effects of an evidence implementation program in clinical practice?’, a semi-structured interview guide was developed. The main open-ended questions designed to achieve the study’s objective were:

- In your perspective, what are the main changes that have occurred with the evidence implementation project (in the development of evidence-based skills and in changes within the team)?
- How do you perceive the knowledge implementation project and the changes that have occurred in the clinical context regarding the use of evidence?
- In your opinion, what are the biggest challenges for clinical practice to be based on the most recent evidence?

The interviews were conducted online using the Colibri^®^ platform, recorded, and transcribed by the researcher who facilitated them.

The interviewer was a member of the research team who was not directly involved in the project. At the beginning of each interview, anonymity and data confidentiality were reiterated. Participants were informed that they could decline to answer any questions and had the right to withdraw from the study at any time.

The interviews lasted an average of 51 min. During the interviews, notes were taken on participants’ facial expressions, response hesitations, management of silent moments, use of paralinguistic features, and other nonverbal communication elements, all of which were considered in the subsequent analysis.

### 2.4. Data Analysis

Categorical content analysis was conducted in a systematic and iterative manner. First, the researchers familiarized themselves with the data through repeated readings of the transcripts, noting initial impressions and ideas relevant to the study aims. All interviews were transcribed verbatim and independently analyzed by two researchers using content analysis techniques [16], supported by the qualitative data analysis software (QDAS) WebQDA^®^.

WebQDA^®^ was selected because it enables structured organization of the dataset, transparent coding and retrieval of excerpts, and collaborative analytic work within the research team, thereby supporting the validation and refinement of the analysis. Initial coding involved identifying meaning units and assigning codes to relevant segments of text. Codes were then compared across transcripts and collated into provisional categories, with attention to patterns of convergence and divergence among participants. Categories were subsequently reviewed and refined against the full dataset to ensure internal coherence, distinctiveness, and alignment with the data. Discrepancies between analysts were discussed and resolved by consensus, and the coding framework was iteratively refined throughout the analytic process. Finally, the categories/themes were clearly defined and labeled, and illustrative excerpts were selected to support interpretation and reporting of the findings.

### 2.5. Study Rigor

The rigor of this study was upheld from its design through all methodological procedures. The same researcher conducted and transcribed all interviews, ensuring consistency. The transcripts were carefully reviewed to maintain trustworthiness. The research team included experienced qualitative researchers.

The findings were analyzed by two researchers and systematically validated by an additional two to ensure the categories’ comprehensiveness, relevance, homogeneity, representativeness, and exclusivity. Constant comparison of findings and assigned codes ensured objectivity and stability in coding. Any modifications to the coding process and their justifications were documented to uphold the study’s methodological transparency and adherence to inductive research principles. After defining the categories, the interpretation conducted by the researchers was returned to the participants for validation of the analysis and interpretation.

The issues highlighted by Colorafi and Evans [13] regarding objectivity (confirmability) were addressed to ensure relative neutrality and reasonable freedom from researcher bias through the following procedures: providing an explicit and detailed description of the study’s methods and procedures; sharing the sequence of data collection, analysis, and presentation methods to create an audit trail; reporting personal assumptions and potential biases; and retaining study data while making it available to collaborators for evaluation.

### 2.6. Ethical Considerations

The study was approved by the Hospital Ethics Committee (Parecer 09/2019 HVFX). Ethical and formal principles were upheld, from institutional authorization allowing nurses to participate in the project to obtaining informed and voluntary consent. In transcribing the interviews, participants’ identities were omitted, and each was assigned a code consisting of the letter “N” (Nurse) followed by a number (1, 2, 3…), ensuring anonymity and confidentiality of the findings.

The findings are securely stored in a cloud system protected by a username and password, with access restricted solely to the principal researcher.

## 3. Results

Seventeen nurses participated in the interviews, comprising 15 women and 2 men, with an average age of 38.7 years and an average of 17.2 years of professional experience. Among them, 7 held a bachelor’s degree, 3 had a postgraduate specialization in nursing, and 7 had obtained a master’s degree.

From the content analysis of the interviews, the following categories emerged:

1. Cultivating a spirit of doubt and fostering an Evidence-Based Practice (EBP) culture.
2. Critically appraise established practices and evidence-based recommendations.
3. Integrating evidence into clinical expertise and driving change by improving outcomes and enhancing quality of care.

### 3.1. Cultivating a Spirit of Doubt and an EBP Culture

Participants observed that the project facilitated a shift in the organizational culture regarding the use of evidence. Two contextual factors identified as key enablers of change were the partnership with an academic institution and the involvement of academics and students in the projects, as highlighted by these two nurses:

*“The fact that there are a partnership and an agreement between the institution’s leadership and the nursing school allows the school to come into the institution. There is a dialogue between the institution and the school, and among the institution, the school, and the healthcare services. Objectives are set for the project and for the students’ involvement (…) so there must be communication, there must be partnership, and the defined objectives must be met. There are joint meetings, and we keep discussing things together.”*

(N11)

*“Having students involved in projects is always an asset because they are more attuned to investigating and addressing new situations that arise, applying knowledge recently acquired at school. Moreover, mentoring students while simultaneously engaging them in projects, in my view, fosters self-awareness, adaptability, decision-making, and overcoming challenges.”*

(N2)

The change process required valuing knowledge, selecting the best available evidence, and implementing it in practice settings. The key to this transformation was team involvement and the redefinition of team communication, shifting its focus toward the application of knowledge to solve complex clinical practice issues. This approach fostered openness and mobilization toward the collective development of new strategies and practices, as described by the following participants:

*“Looking back to 2014, when we started, there is now a heightened awareness among all team members regarding the importance of using evidence to solve problems and even to create learning opportunities for students (…). Back then, there was tremendous entropy—we had outstanding professionals who mastered their field, perhaps better than many others, yet knowledge was not shared. That was a missed opportunity. However, bringing the team closer together and fostering more dialogue made all the difference.”*

(N7)

*“(…) it has a lot to do with the environment in which one is embedded. Either there is a group with a mindset of moving forward, making things happen, and incorporating others into that momentum, or, if that drive is lacking, everything stagnates. I believe there must be focus, a collective willingness, not just from an individual but from the group. Because conducting research alone… no one embarks on that journey by themselves, in my opinion.*

(N1)

The participants believe that fostering an organizational culture permeated by evidence also necessitates the creation of dedicated moments for sharing and identifying issues that can be addressed using knowledge. These moments serve to analyze the entire process, from problem identification to its partial or complete resolution. In their perspective, these opportunities for sharing foster meaningful learning experiences and provide the motivation to continue investing in EBP projects. Simultaneously, they enable the planning of work in alignment with appropriate objectives and facilitate the evaluation of contributions to the project’s development process.

*“From my point of view, it was a two-in-one experience because guiding students while having them involved in our projects promoted self-awareness, adaptability, decision-making, and the ability to overcome difficulties.”*

(N8)

*“Ah! These moments are primarily about discussing what can be changed and sharing knowledge, allowing us to understand why care was provided in a certain way and how we can adjust it by justifying what might be the best approach. We do not engage in this discussion extensively during shift handovers due to time constraints. However, having these moments during team meetings has been beneficial in creating the habit of identifying solutions and assessing outcomes.”*

(N3)

Content analysis of the findings highlights the importance of allocating time and space for multidisciplinary team meetings, fostering communication, and promoting collective learning among students and faculty. The analysis of results through the lens of evidence played a central role in encouraging professionals to question existing practices and seek solutions by leveraging specialized knowledge, research, and negotiation. A progressive shift in service dynamics was observed, particularly in problem-solving, solution-seeking, and evidence-informed decision-making.

### 3.2. Critically Appraise Established Practices and Evidence-Based Recommendations

A fundamental component of effectively utilizing evidence is the professional competence to critically evaluate research findings, clinical guidelines, and practice norms regarding their validity, impact, and applicability to the specific clinical context. These skills necessitate enhancing professionals’ scientific literacy, enabling them to identify the best available evidence through database research, assess study quality, and compare recommendations with current clinical practices in their specific work environment.

As these nurses argue:

*“I believe there is still a significant gap between knowledge production and the subsequent implementation of interventions based on the best available evidence (…) Discussions happen, and everyone contributes in their way, but there is always someone who takes the initiative to research. ‘Do you remember that conversation we had? Well, I looked it up, and this is what the research says.’ (…) Although we now have Google, where we can conduct various searches, not everything we find is accurate; we cannot blindly trust the validity of what we encounter. However, I believe that scientific documents exist, and we can access them—we must not act without grounding our practice in solid evidence. If I have a question about a procedure or if something new arises, I should have the skills and ability to investigate why I am implementing that technique, that practice, that approach, those protocols…”*

(N8)

*“When we can attribute meaning to our knowledge and organize acquired information and developed skills, they become facilitators of knowledge application. Whenever strategies are developed and integrated to retain acquired information, we will always be capable of using that knowledge to solve various problems, making acquired knowledge a facilitator for its own use.”*

(N6)

Healthcare professionals recognize that the dialog between current practices and optimal practices necessitates continuous learning, skill development, and negotiation.

*“I consider it crucial to select knowledge that addresses or resolves any health-related issue for any patient or family. This way, healthcare professionals step out of their comfort zones and seek new pathways of understanding, utilizing scientific instruments to achieve the best possible clinical evidence. Although this concept is frequently mentioned in our discourse, in reality, it requires us to learn, acquire knowledge, question, seek evidence, and negotiate among ourselves on how we will modify our practices.”*

(N17)

*“We are discussing real-life examples… For instance, this week, I had the opportunity to review several opinions from the specialty board to understand their position on motor rehabilitation for Long COVID patients. What could we do? What was permitted? I immediately shared my findings with my colleagues, meaning there is now greater openness—I found this knowledge, so I will share it, and we will discuss it and, if necessary, translate it into practice.”*

(N2)

The movement toward embracing new practices stems from the critical appraisal of evidence, dialog, and the collective negotiation of pathways. There is co-management in constructing new practices, rather than an imposed, meaningless implementation, even though each professional may have different timelines for assimilating and applying new work tools. The team values diverse knowledge and practices as essential assets in addressing challenges effectively.

Based on the above, it can be inferred that the existence of structured moments allowing professionals to analyze their “ways of doing” and compare them with evidence-based recommendations is crucial for negotiation, team adherence to the introduction of new interventions, practice changes, and the development of competencies for seeking, reading, and analyzing scientific literature.

### 3.3. Integrating Evidence into Clinical Expertise and Driving Change to Enhance Outcomes and Improve the Quality of Care

The analysis of the interviews highlights the shared responsibility and involvement of the patient and their family with the healthcare team in care provision, as well as the importance participants place on observing the outcomes of implementing research findings in clinical settings. It is the positive impact on patient functionality, the reduction in average length of hospital stays, and the prevention of complications that motivate nurses to enhance clinical decision-making based on evidence.

*“For instance, the multidisciplinary discharge planning meetings, where we involve the patient, the family, and take into account who the patient considers a key reference during education and training moments. Additionally, having the opportunity to establish telephone contact with community healthcare units when we identify a situation that may be more vulnerable and require a faster intervention from community colleagues. This was not done before, but as we implemented the project, we noticed that patients and families were better prepared at discharge, with less anxiety about returning home because they had already been in contact with community colleagues—often with a scheduled visit—and this was highly motivating for the team because we could see tangible results.”*

(N11)

*“Our hospital stays are significantly shorter because we have a more structured organization and a much more thorough discharge planning process. Even in daily practice, we now have access to additional resources that enrich the service in terms of monitoring, rehabilitation, and other aspects. The benefits are undeniable. In numerical terms, our length of stay has decreased significantly, and care delivery is much more organized.”*

(N15)

*“I believe so; they observe improvements in their practice (…) This serves as motivation and encourages them to base their practice on evidence.”*

(N16)

When asked whether the project influenced attitudes toward implementing evidence in clinical practice, the professionals responded:

*“Yes, absolutely. The pursuit of knowledge to solve any given problem makes professionals more aware of different situations and enables them to intervene earlier to resolve them.” (N9) “Witnessing improvements at a time when patients and communities present increasingly complex clinical challenges—demanding higher-quality and safer responses from healthcare professionals and organizations—requires enhanced effectiveness, efficiency, and impact in health interventions. This is precisely what we have observed over the past five years, and it is highly motivating to continue. Since implementing this project, I believe we have achieved extraordinary outcomes.”*

(N10)

*“Yes, without a doubt, because the benefits are highly tangible and visible. Those who knew the service before and see it now can clearly recognize the improvement in the quality of care delivery.”*

(N12)

The value of EBP projects and the rationale for their continuity and sustainability are closely linked to predefined indicators assessed semi-annually, demonstrating measurable improvements in team health outcomes, nursing care outcomes, and the economic benefits associated with these indicators.

## 4. Discussion

Authors highlight one of the barriers to EBP as the organizational culture that rewards task-based and routine practices, which, either directly or indirectly, influences attitudes towards the use of evidence, often devaluing it. Our findings suggest that changing the organizational culture can be achieved through specific projects involving senior managers and engaging them with organizational policies that promote EBP, focusing on the implementation of results in clinical practice [17].

For its implementation, continuous support and stimulation of EBP seem to be prerequisites for developing and sustaining an Evidence-Based Nursing (EBN) culture that ultimately contributes to enhanced quality in fundamental nursing care [18]. An additional important aspect is the role of leaders. Leaders have the responsibility to engage staff at all levels, support an EBP culture, and allocate resources to provide the necessary infrastructure that promotes clinical decision-making based on the best available evidence [2,18].

Another study suggests that the level of organizational readiness is the greatest factor in EBP implementation [19]. According to these authors, during the initial process of introducing and establishing EBP, nursing organizations will need to minimize anticipated barriers, enhance facilitators, and work towards building an infrastructure that includes a clear vision, policy-making, budgeting, skilled personnel, and excellent facilities within the organization [18]. Additionally, investment in educational training is necessary to improve knowledge, develop skills to critically assess evidence, and facilitate the transfer of knowledge to clinical settings [11,19].

The connection between nursing care and quality outcomes is indisputable, but for nursing’s future within the quality movement, it is essential that leaders commit to incorporating evidence into nursing practice [2,7,11,19]. Our study did not explore the processes of leadership and the characteristics of leaders necessary to promote this change in organizational culture. Future studies should explore this aspect.

Nurses perceive that their attitudes towards EBP have changed. These findings corroborate those of Lovink et al., [18] who concluded that nursing teams became more open, critical, and reflective, and they integrated EBP into daily practice in tailored and creative ways.

Within the category of Critically appraise established practices and evidence-based recommendations, the findings suggest that professionals are progressively constructing understandings about how to engage with evidence in practice. These understandings appear to emerge through attention to the individual’s/family’s context and culture—within a person centered care framework—as well as through consideration of the team’s and healthcare system’s possibilities and constraints, particularly regarding communication and inter-service collaboration. This aligns with the expectations for operationalizing competence from a holistic/dialogical perspective, where the professional resolves problems by mobilizing their abilities in various ways, considering the context, culture, ethical attitude, and socially defined standards of excellence [20].

There is also an emerging need for the development of professional competencies at multiple levels that enable professionals to enhance their scientific literacy. Fisher et al. advocate for the use of a “top-down” strategic plan and a “bottom-up” competency-based approach to sustain EBP development, employing a novice-to-expert competency framework that encourages each nurse to embark on their individual journey of integrating EBP into daily practice [20]. Fu et al. emphasize that to improve nurses’ EBP competency, it is not sufficient to acquire knowledge alone through education and training; it is also essential to apply and demonstrate EBP knowledge in clinical practice [21].

The enhancement of competencies in evidence transfers, situation assessment, and evidence implementation should be fully embraced and promoted within clinical settings [1,2,7,21,22]. The modified Policy Delphi study conducted by Saunders, Gallagher-Ford, and Vehviläinen-Julkunen [22] suggests that a fundamental understanding of the core principles of EBP and the skills to apply the steps of the EBP implementation process to daily practice are essential competencies for all healthcare professionals, including nurses, to enable their systematic engagement in EBP.

The final category, Integrate evidence in clinical expertise and promote change by improving outcomes and increasing the quality of care, reinforces the importance of the systematic implementation of EBP in daily care delivery to achieve enhanced care quality and improved patient outcomes [22]. It is expressed in the voices of the participants that the recognition that the implementation of evidence in clinical practice leads to benefits for patients, particularly in terms of patient functionality, reduced average hospitalization time, and the prevention of complications, serves as a major motivation to continue such projects and further promote evidence-based clinical decision-making.

The transfer of knowledge into clinical practice and its ongoing support allow for the achievement of the triple aim by enabling the simultaneous pursuit of improvements across three areas: population health outcomes, quality of care, and value for the system [23]. However, we align with the authors’ viewpoint who observe that its implementation remains challenging [7,11,23,24,25,26,27] due to time constraints, insufficient staffing, financial limitations, resource shortages, lack of teamwork, and inadequate organizational support [24]. Additionally, the results of studies may not be generalizable to nurses’ settings, facilities may be insufficient, and physicians may not cooperate with the implementation [27].

The EBP interventions should not remain isolated initiatives; they need to be scaled systematically across organizations, underpinned by structured leadership support that secures resources and builds staff commitment. In addition, partnerships with academic institutions, should be formalized to ensure ongoing access to continuing professional development, research support, and mentorship [4]. Educational interventions and supportive work environments are recommended to enhance EBP engagement across all levels of nursing practice [26].

### 4.1. Implications for Practice

This study highlights the need for healthcare institutions to shift their organizational culture, contributing to evidence-supported decision-making. Some emerging suggestions include the creation of opportunities for team discussions to identify problems and engage in their resolution, analyzing existing practices, confronting these practices with evidence-based recommendations, and investing in the training of professionals to develop competencies in EBP.

### 4.2. Limitations of This Study

This study is subject to limitations associated with the methodology, participants, data collection techniques, and their subsequent analysis. The decision to conduct a qualitative study within a specific context limits the transferability of the findings. Although this study explores the perspectives on the effects of an evidence-implementation program in clinical practice, the participants had been involved with the project from its inception, and personal experiences related to their involvement may have influenced their perceptions of its results.

The use of a semi-structured interview guide, which allows for flexibility and enhances the richness and depth of findings, also increases the difficulty of conducting the interviews and introduces greater diversity in responses. The involvement of an experienced researcher in conducting semi-structured interviews and qualitative studies likely helped mitigate potential bias.

Although the analysis was conducted by two researchers and validated by the team and participants, it may not have been entirely free from bias.

The study was conducted in a specific organizational context and involved participants of a particular project, which may limit the transferability of its findings to other settings. As a result, the findings should be interpreted with caution, since their direct transfer to other contexts may be constrained by differences in organizational structure, resources, leadership arrangements, professional roles, and local cultures of practice. Nevertheless, by elucidating key processes and contextual influences underpinning the phenomenon under investigation, the analysis and discussion provide insights that are likely to be relevant beyond the immediate case and may help inform practice development and future research in similar organizational and professional contexts.

## 5. Conclusions

This qualitative study, involving 17 nurses, sought to explore how participants perceived the effects of their involvement in evidence-implementation programs on their nursing practice. Through content analysis of semi-structured interviews, nurses described the need for a shift in organizational culture—one that creates time and space for teams to critically reflect on practice, recognize gaps between theory and clinical realities, engage with research, and translate knowledge into care settings.

From the participants’ perspectives, communication and structured opportunities for dialog and shared reflection were central to examining clinical practices and fostering the development of EBP competencies. Nurses also emphasized that a significant source of motivation for implementing evidence was their perception that it produces concrete and meaningful benefits for healthcare service users.

## Data Availability

Data can be obtained by contacting the corresponding author.

## Abbreviations

The following abbreviations are used in this manuscript:

EBP	Evidence-Based Practice
N	Nurse
WHO	World Health Organization
